# NOTCH receptors in gastric and other gastrointestinal cancers: oncogenes or tumor suppressors?

**DOI:** 10.1186/s12943-016-0566-7

**Published:** 2016-12-09

**Authors:** Tingting Huang, Yuhang Zhou, Alfred S. L. Cheng, Jun Yu, Ka Fai To, Wei Kang

**Affiliations:** 1 Department of Anatomical and Cellular Pathology, State Key Laboratory of Oncology in South China, Prince of Wales Hospital, The Chinese University of Hong Kong, Shatin, N.T, Hong Kong, SAR People’s Republic of China; 2 Institute of Digestive Disease, Partner State Key Laboratory of Digestive Disease, The Chinese University of Hong Kong, Hong Kong, SAR People’s Republic of China; 3 Li Ka Shing Institute of Health Science, Sir Y.K. Pao Cancer Center, The Chinese University of Hong Kong, Hong Kong, SAR People’s Republic of China; 4 Shenzhen Research Institute, The Chinese University of Hong Kong, Shenzhen, People’s Republic of China; 5 School of Biomedical Sciences, The Chinese University of Hong Kong, Hong Kong, People’s Republic of China; 6 Department of Medicine and Therapeutics, The Chinese University of Hong Kong, Hong Kong, People’s Republic of China

**Keywords:** Gastric cancer, Notch pathway, NOTCH receptors

## Abstract

Gastric cancer (GC) ranks the most common cancer types and is one of the leading causes of cancer-related death. Due to delayed diagnosis and high metastatic frequency, 5-year survival rate of GC is rather low. It is a complex disease resulting from the interaction between environmental factors and host genetic alterations that deregulate multiple signaling pathways. The Notch signaling pathway, a highly conserved system in the regulation of the fate in several cell types, plays a pivotal role in cell differentiation, survival and proliferation. Notch is also one of the most commonly activated signaling pathways in tumors and its aberrant activation plays a key role in cancer advancement. Whether Notch cascade exerts oncogenic or tumor suppressive function in different cancer types depends on the cellular context. Mammals have four NOTCH receptors that modulate Notch pathway activity. In this review, we provide a comprehensive summary on the functional role of NOTCH receptors in gastric and other gastrointestinal cancers. Increasing knowledge of NOTCH receptors in gastrointestinal cancers will help us recognize the underlying mechanisms of Notch signaling and develop novel therapeutic strategies for GC.

**Electronic supplementary material:**

The online version of this article (doi:10.1186/s12943-016-0566-7) contains supplementary material, which is available to authorized users.

## Background

GC is the fifth most common cancer types globally and the second leading cause of cancer death [1]. The relatively high mortality is mainly because of its silent nature, late clinical presentation and genetic heterogeneity [2]. The potential risk factors include *Helicobacter pylori* (*H. pylori*) or Ebstein-Barr virus (EBV) infection, high-salt and low-vegetable diet, smoking, chronic gastritis with glandular atrophy and intestinal metaplasia, and host genetic susceptible single nucleotide polymorphisms (SNPs) [3]. Histologically, Lauren classification divides GC into intestinal and diffuse types, accounting for 54% and 32% respectively [4]. Intestinal GC is strongly associated with *H. pylori* infection and often preceded by intestinal metaplasia, while diffuse type exhibits poor differentiation and early metastasis with unfavorable outcome. In The Cancer Genome Atlas (TCGA) study, GC is clustered into four molecular subtypes: EBV positive (9%), microsatellite instability (MSI) (22%), genomically stable (GS) (20%), and chromosomal instability [5] (50%) [6]. The poor prognosis of GC is mainly related to the limited understanding of its etiological factors and pathogenesis model. GC can be attributed to deregulation of signaling pathways, which are often followed by precancerous lesions. Meanwhile, the challenges of GC treatment contain novel strategies for early GC detection and precision therapies for GC patients. Therefore, a better understanding of the deregulated signaling pathway in GC is essential for the development of new therapeutic drugs.

GC is proposed to derive from the complex interplay of genetic, epigenetic and environmental factors that deregulates potential oncogenic signaling pathways [7–9]. Moreover, it is generally believed that gastric carcinogenesis is due to dysfunction of oncogenic cellular pathways, such as Wnt/β-catenin, nuclear factor-κB, Hedgehog, Notch and epidermal growth factor receptor (EGFR) pathway [10]. Activation of these signaling cascades leads to the acquisition of malignant phenotypes including increased cell proliferation, evasion of apoptosis and enhanced invasiveness. Among these pathways, Notch signaling is involved in direct cell-cell communication, thereby controlling cell differentiation, proliferation and apoptosis [11]. Aberrant Notch signaling activation has been implicated in a variety of cancers. Mechanism of how NOTCH receptors impact gastric cell transformation remains enigmatic, because NOTCH receptors seem to behave as either oncogene or tumor suppressor depending on different cancer types (Table 1). Different expression levels and signaling cascades of NOTCH receptors may be a reason to explain their distinct functions. In this review, we summarize the published data regarding to the role of NOTCH receptors in gastrointestinal tumors and provide the evidence for their involvement in tumorigenesis, especially in GC. Improved knowledge of NOTCH receptors and Notch signaling cascade will help to elucidate the molecular mechanisms and develop novel therapeutic strategies for GC.

**Table 1:** Summary of NOTCH receptors in gastrointestinal malignancies

NOTCH receptor	Cancer Types	Functions	Mechanism	References
NOTCH1	Gastric cancer	Oncogene	Activated NOTCH1 promotes cell proliferation, metastasis and inhibits cell apoptosis. NOTCH1 maintains the cancer stem-like properties in diffuse type gastric cancer through RBP-Jκ dependent pathway.	[48, 55–60]
		Tumor suppressor	Increased NOTCH1 expression up-regulates PTEN.	[64]
	Hepatocellular carcinoma	Oncogene	Activated NOTCH1 promotes cell invasion through the regulation of PTEN and FAK. Up-regulation of NOTCH1 increases Snail expression and functions as endothelial progenitor cells to initiate tumor vasculogenesis.	[80–82, 89–91]
		Tumor suppressor	NOTCH1 induces degradation of the Snail protein and inhibits Snail-induced cell invasion. Through E2F transcription factors, Notch pathway activation forms a negative feedback loop to inhibit HCC proliferation.	[83, 84]
	Colorectal cancer	Oncogene	Activated NOTCH1 represses p27 to promote cell cycle and proliferation. Moreover, it induces proliferation through the activation of cyclin D1 and Hes1 and increases the stemness related proteins expression.	[106, 109–111]
		Tumor suppressor	NOTCH1 activation suppresses the expression of WNT-targeting genes.	[112]
	Esophageal cancer	Oncogene	High NOTCH1 expression is associated with poor survival and promotes the growth of EAC cells. Also, it is involved in the maintenance of EAC cancer stem cells and increases the invasion and metastasis of ESCC cell line EC-9706.	[19, 125–127]
		Tumor suppressor	Activated NOTCH1 inhibits cell proliferation and induces cell apoptosis in ESCC.	[124]
	Pancreatic cancer	Oncogene	Activated NOTCH1 promotes cell proliferation, migration, and metastasis. Furthermore, NOTCH1 overexpression inhibits apoptosis and leads to EMT phenotype.	[132, 134]
		Tumor suppressor	NOTCH1 exerts tumor-suppressive function in a model of K-RAS-induced pancreatic ductal adenocarcinoma.	[135]
NOTCH2	Gastric cancer	Oncogene	NOTCH2 induces COX-2 expression to enhance gastric cancer progression. NOTCH2 and miR-23b interplay to form a reciprocal regulation loop in gastric carcinogenesis.	[65, 67]
		Tumor suppressor	NOTCH2 suppresses cell invasion through inhibition of the PI3K/AKT signaling pathway.	[68]
	Hepatocellular carcinoma	Oncogene	NOTCH2 signaling promotes the proliferation and tumor formation of HCC cells, and confers aggressive behavior and immature morphology in human HCC cells. NOTCH2 activation levels are consistent with clinical severity and prognosis of HCC patients.	[95–98]
	Colorectal cancer	Tumor suppressor	NOTCH2 decreases tumor differentiation and predicts better survival.	[113–115]
	Pancreatic cancer	Oncogene	NOTCH2 activates Myc signaling.	[136]
NOTCH3	Gastric cancer	Tumor suppressor	NOTCH3 contributes to glandular differentiation associated with MUC2 and MUC5AC expression.	[69]
	Hepatocellular carcinoma	Oncogene	NOTCH3 expression enhances aggressive traits in HCC and plays a crucial role in HCC progression by interacting with β-catenin. NOTCH3 silences p53 in HCC.	[99, 100, 157]
	Colorectal cancer	Oncogene	NOTCH3 promotes tumor growth, tumor proliferation and migration through up-regulating MSI-1 expression.	[116–119]
	Esophageal cancer	Tumor suppressor	NOTCH3 contributes to esophageal cell fate decisions and inhibits TGF-β-mediated EMT through ZEBs.	[128]
	Pancreatic cancer	Oncogene	NOTCH3 promotes cell proliferation and activates PI3K/AKT pathway.	[138]
NOTCH4	Gastric cancer	Oncogene	NOTCH4 promotes GC growth through the activation of Wnt1/β-catenin signaling.	[70]
	Hepatocellular carcinoma	Oncogene	NOTCH4 overexpression might serve an independent prognostic factor of shorter disease specific survival.	[88, 102]

### The main components of Notch signaling pathway

*NOTCH*, which was cloned in the mid-1980s, encodes a receptor with a single transmembrane domain [12, 13]. With evolutionarily conserved property, Notch signaling pathway is initiated by receptor-ligand interaction between two neighboring cells, wherein a membrane-tethered NOTCH ligand on one cell interacts with the other cell that presents a NOTCH receptor. The extracellular domain of NOTCH receptor contains epidermal growth factor-like (EGF) repeats that contribute to ligand binding [14]. Mammals possess four NOTCH receptors (NOTCH1-4) and five typical ligands with DSL (Delta/Serrate/LAG-2) domain named Delta-like (DLL) 1, 3 and 4, JAG1 and JAG2. In addition, there are some atypical ligands including DNER, F3/Contactin, and NB-3 without DSL domain. Upon ligand binding, NOTCH receptors undertake two proteolytic cleavage processes. The first cleavage occurs extracellularly and is close to the transmembrane domain [15]. Furthermore, the second cleavage is catalyzed by γ-secretase [16]. NOTCH intracellular domain (NICD) is released by the second cleavage and subsequently translocated into the nucleus [17, 18]. NICD cannot directly combine with DNA but heterodimerizes with the DNA-binding protein CSL (CBF-1/Suppressor of Hairless/Lag-1) to activate transcription of genes containing CSL binding sites. In the absence of NICDs, CSL inhibits Notch-targeting genes. In the presence of ligand, NICDs are released and bind with CSL to subsequently recruit a coactivator complex for activating transcription of Notch-targeting downstreams.

CSL is a transcriptional repressor associated with a SMART complex, which binds to the consensus DNA sequence during the absence of NICDs. The binding of NICDs with CSL results in the activation of two families of the best characterized Notch-targeting genes [Hairy enhance of spilt (HES) and Hairy/Enhancer of Spit related with YRPW motif [19–21]. HES/HEY family members repress the transcription of tissue specific differentiation factors, therefore, Notch signaling pathway leads to the maintenance of stem or progenitor cells through the inhibition of differentiation [22].

### The modulation of Notch signaling pathway

Notch signaling pathway is regulated at the transcriptional or post-transcriptional levels. A previous study confirmed Notch is negatively regulated by distinct miRNAs [23]. Additionally, the ubiquitination pathway is important to Notch signaling activity, because E3 ubiquitin ligases regulate the amount of NOTCH receptors and other components, which inhibits Notch activity [24]. The ligand-receptor interactions are modulated by post-translational modification of NOTCH receptors. The extracellular EGF repeats of NOTCH receptors are modified by *O*-glucose or *O*-fucose additions. This process is mediated by Fringe family glycosyltransferases [25]. Therefore, the relative binding activity of ligand-receptor pairs can be adjusted, thus to promote the activation of Notch signaling cascades [26]. Numb and Numbl are docking proteins and function as cytoplasmic Notch signaling inhibitors [27], helping to remove NOTCH receptors from the cell membrane and degrade them. However, Numb translation is repressed by MSI1, which further activates Notch signaling [28]. Phosphorylation of NICDs on serine residues promotes the formation of NICD/CSL complex and is responsible for the intracellular localization of NICDs [29]. Moreover, the Cyclin/Cdk pair strongly elevates NICD phosphorylation which contributes to NOTCH activation [30].

### The physiological role of NOTCH receptors in gastrointestinal tract

NOTCH1-3 receptors, as well as DLL1, JAG1 and JAG2 ligands, are differentially expressed throughout gastroenterological tract [31]. Besides, they are not only expressed variously in proliferative and post-mitotic cells in adult rat gut, but also in the epithelial, immune and endothelial cells [31]. Under normal physiological conditions, Notch signaling plays a fundamental role in cell fate determination in nearly all developing tissues and organs [32]. In addition, it regulates gastrointestinal stem cell proliferation and differentiation. In inducible gut-specific NOTCH-mutant mice, Notch signaling controls gut crypt differentiation and proliferation and is involved in the regulation of cell cycle progression of crypt progenitor cells [33].

High expression of NOTCH3 and JAG2 is found in gastric fundus with low expression of DLL1. In the stomach body region, expression of NOTCH2, NOTCH3, JAG1 and JAG2 is also markedly abundant. NOTCH1-3 and HES1 are expressed in human gastric mucosa [34]. Gastric epithelium is continuously regenerated by gastric stem cells, which give birth to parietal cells, chief cells, surface mucous cells, mucous neck cells, and enteroendocrine cells. The adult mammalian gastric epithelia renew themselves continually through the activity of stem cells that locate in the isthmus of individual gland units. Notch signaling is required to keep the gastric stem cell compartment [35] and monitors the proliferation and differentiation of stem cells as well as gastric tissue growth, while uncontrolled Notch activity in stem cells leads to polyp formation [36]. Recently, Notch signaling is suggested as a key regulator of self-rehabilitation and differentiation of Lgr5 antral stem cell [36].

In mouse esophagus, expressions of NOTCH1, NOTCH2, JAG1 and JAG2 is highly detected in the basal layer [31]. In the human esophagus, esophageal epithelial stem cells are in the bottom of the basal layer, where Notch signaling is activated to regulate the balance of stem and progenitor cells [37]. Notch signaling inhibition in mouse esophagus induces deregulated squamous cell differentiation and aberrant basal cell proliferation [38].

In early liver development, Notch signaling also plays an important role in cell fate decision. In NOTCH2 and JAG1 heterozygous mice, bile duct paucity is found in mutant mice [39]. The role of Notch signaling has also been demonstrated in the liver regeneration, where it is sufficient to reprogramme hepatocytes into endothelial biliary cells [40].

In the intestine, Notch cascade controls cell proliferation and differentiation [41]. DLL1 is the most important ligand for the NOTCH1 receptor in intestinal crypt epithelium and the absence of DLL1 causes an increase of goblet cells [42]. NOTCH1-3 are highly expressed at the basal crypt of the human colon, while at the top of the crypts, there is a profusion of JAG1 [43]. Notch plays a vital role in the maintenance of normal intestinal epithelia and is essential for regulating differentiation of colonic goblet cells and stem cells [44]. The innermost layer of the colon is composed of stem cells. The colon contains a gradient of signaling pathways including Wnt, Hh, BMP and Notch [45]. Notch and Wnt signalings are activated at the base of the crypt, a place where these signaling pathways work together to regulate the stem cell regeneration, proliferation and differentiation. There are two mechanisms of Notch pathway in the intestine. One is the maintenance of the stem cell pool through negative regulation prevents the differentiation of stem cells. The other is to manage the balance between absorptive and secretory lineages through promoting differentiation in one direction while suppressing the other possible outcomes [46].

### The deregulated NOTCH receptors in gastrointestinal cancers

Deregulated Notch cascade was first identified in T-cell acute lymphoblastic leukemia (T-ALL). NOTCH mutations were suggested to be associated with specific forms of leukemia [47]. Subsequently, links between NOTCH and tumors are extending to multiple cancer types. The role of NOTCH in solid tumors is likely to highly context dependent and its functions seem sometimes controversial. In this part, we will comprehensively review the functional role of NOTCH receptors in gastrointestinal cancers.

#### Gastric adenocarcinoma

The abnormal richness of NOTCH1-4 mRNA was found to be associated with unfavorable overall survival for 876 GC patients for 20 years [48]. In GC, activated NOTCH1 was a poor prognostic factor for patients [49]. Also, increased NICD1 was observed in tumor dedifferentiation, depth of tumor invasion, lymph node metastasis, surface morphology and Lauren classification [50]. In GC cell lines, DLL1 expression is epigenetically regulated by promoter methylation although DLL1 activates Notch1 signaling pathway. Aberrant DLL1 promoter hypermethylation has been showed in 52% primary tumors in at least one region but not in healthy controls. Therefore, epigenetic regulation of the NOTCH ligand DLL1 only partly explained the activated NOTCH1 signaling in GC [51, 52]. NOTCH1 expressed in both premalignant and cancer tissues, especially in samples of intestinal metaplasia and well-differentiated intestinal type. It may be crucial in both promoting the metaplastic transition of gastric epithelial cells and maintaining a constant proliferation of internalized epithelial cells [53, 54]. Over-activated NOTCH1 was considered to prevent gastric carcinoma BGC-823 cells from TNFα-induced apoptosis [55]. Apart from faciliating GC progression via cyclooxygenase-2 (COX-2) [56], activation of the NOTCH1 signaling was also suggested to be related with metastasis of human malignancies [57]. Moreover, over-expressed NOTCH1 enhanced interaction between nuclear STAT3 and Twist promoter and activated NOTCH1/STAT3/TWIST signaling axis, further to promote GC progression [58]. Meanwhile, NOTCH1 silencing reduced proliferation and invasion in SGC-7901 GC cells [59]. A group of scientists pointed out that NOTCH1 acted as a significant part in the maintenance of the cancer stem-like phenotype of diffuse type GC through a RBP-Jƙ binding motif in the 5′ promoter region of *CD133* gene. They also suggested NOTCH1 inhibition might serve as an effective therapy against CD133-positive diffuse type GC [60]. Some reports also suggested NOTCH1 regulatory mechanisms by some tumor-suppressive miRNAs, such as miR-34 family [61], miR-124 and miR-935 [58, 62]. All these miRNAs have been proved to repress NOTCH1 expression during GC progression. Meanwhile, NOTCH1 pathway, together with miR-151-5p, interplayed with p53 to form a reciprocal regulation loop in controlling gastric carcinogenesis [63]. However, there was a paper reported anti-tumor role of NOTCH1 in GC. Zhou W et al. demonstrated that NOTCH1 was absent or minimally expressed in GC tissues but abundant in paired normal gastric mucosa. Sequentially, they highlighted a novel AKT1/NF-ƙB/NOTCH1/PTEN axis as a key mechanism of chemoresistance in GC [64]. In addition, the active intracellular domain of NOTCH2 binds with COX-2 promoter region and induces COX-2 expression [65]. These findings implied that NOTCH1 and NOTCH2 boosted GC carcinogenesis through up-regulating COX-2. High NOTCH2 expression was identified as a prognostic parameter, as it was correlated with poor survival in GC patients [66]. Recently, there were results also revealing the NOTCH2 regulation by miR-23b in GC [67]. Contradictorily, tumor suppressive role of NOTCH2 was also reported in one publication. The authors stated that NOTCH2 decreased cell invasion through the PI3K/AKT pathway in MKN45 cells [68]. NOTCH3 profusion was found in the intestinal type of GC, with a better histological differentiation, indicating its role as a favorable prognostic indicator [69]. NOTCH4 activation promoted GC growth through the overexpression of Wnt1, β-catenin and downstream target genes, c-Myc and cyclin D1 [70].

**Fig. 1:**
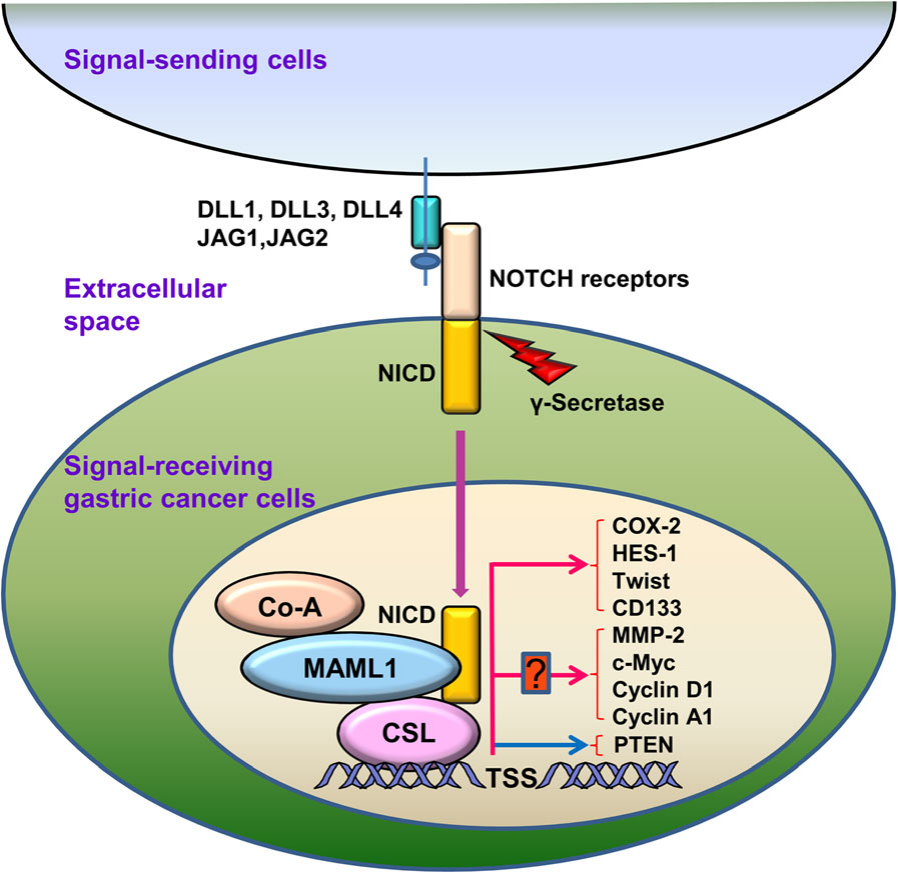
Schematic representation of Notch signaling cascade in GC cells. Notch signaling is initiated by ligand (DLL1/3/4 or JAG1/2) binding to NOTCH receptors (NOTCH1-4). Then a series of proteolytic cleavages occur, resulting in the release of NICDs. NICDs are translocated to the nucleus and bind with CSL to activate the expression of Notch downstream targets. The downstream proteins, such as COX-2, HES-1, Twist and CD133, promote cell proliferation, inhibit cell apoptosis and maintain cancer stem-like phenotypes. CSL, C protein binding factor 1/Suppressor of Hairless/Lag-1; NICD, Notch intracellular domain; TSS, transcription start site

Moreover, a study suggested that restraint of NOTCH receptors by two γ-secretase inhibitors (GSIs) suppressed cell proliferation and induced cell apoptosis. DAPT, a γ-secretase inhibitor, diminished GC growth, invasion, metastasis and epithelial-mesenchymal transition (EMT) through NOTCH1 pathway [71]. Additionally, combined treatment with both GSIs and chemotherapeutic agents significantly minimized the orthotopically transplanted gastric tumors in mice [72]. As for the therapeutic strategies, Hyun-Woo Lee et al. pointed out that targeting Notch signaling by GSIs enhanced the cytotoxic effect of 5-FU in GC [73]. There was also another project indicated that the IL-6/STAT3/JAG1/NOTCH axis might be a target for improving the efficacy of trastuzumab in GC treatment [74]. To better under the Notch signaling in gastric tumorigenesis, we summarized the main reports in a schematic presentation (Fig. 1).

**Fig. 2:**
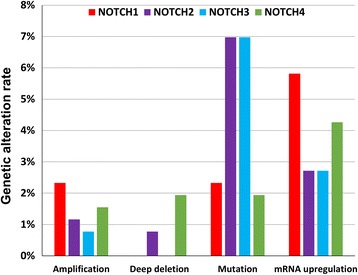
The genetic alteration rates of NOTCH1-4 in GC from TCGA cohort. The bar chart indicates the amplification, deep deletion, mutation, mRNA upregulation rates of NOTCH1-4 in primary GC samples

To further address the Notch signaling cascade in GC with more updated information, we summarized the genetic alteration rates including copy number changes (amplification and deep deletion), somatic mutations and mRNA upregulation of NOTCH1-4 in the TCGA cohort (Fig. 2) [75, 76]. From the TCGA cohort analyzed by cBioPortal, NOTCH2 and NOTCH3 have the highest mutation rate (7% respectively) and mutations include truncating mutation, missense driver mutation and missense passenger mutation. The genomic amplification was merely one of the multiple reasons for high NOTCH1-4 mRNA expression in some GC cases, so we checked the promoter methylation status of NOTCH1-4 in GC. We found the promoter methylation level of NOTCH2, but not NOTCH1, is negatively correlated with its mRNA expression with significance (*P* < 0.001, Additional file 1: Figure S1). As *H. pylori* and EBV infection are the main risk factors for GC, we then checked the expression of NOTCH1-4 with the *H. pylori* or EBV infection status. However, we did not achieve any positive correlation between the expression of NOTCH1-4 with *H. pylori* infection (Additional file 2: Figure S2) or EBV infection (Additional file 3: Figure S3).

#### Hepatocellular carcinoma (HCC)

In addition to GC, aberrant Notch pathway has been linked to liver malignancies. Notch cascade was activated in human HCC samples and promoted hepatic carcinogenesis in mice as previous research showed [77]. To address which NOTCH member took the key position during the progress of liver cancer, Huntzicker et al. used antibodies to specifically target NOTCH1-3 and JAG1 respectively in xenograft mouse model of primary HCC driven by AKT and N-RAS [78]. They found that different NOTCH receptors had drastically different functions during HCC development and inhibition of NOTCH2 represented the most significant therapeutic option in the treatment. Moreover, Wang et al. also proposed a non-proteasome mediated feedback loop between NOTCH1 and Wnt/β-catenin signaling in activating liver cancer stem cells [79]. NOTCH1 activation contributed to tumor cell growth and proliferation while NOTCH1 down-regulation inhibited the invasion and migration by inactivating the Cox-2/Snail/E-cadherin pathway or through regulation of PTEN and FAK [80–82]. However, NICD1 was also demonstrated as a tumor suppressor gene in HCC. It induced the degradation of Snail protein by ubiquitination and inhibited Snail-induced cell invasion [83]. During tumor progression, Notch signaling exerted a tumor-suppressive role through feedback loop in response to E2F transcription factors activation in *Rb*-family-triple-knockout liver cells [84]. Also, Pofut1 overexpression accelerated the cell proliferation and migration in HCC through the activation of Notch pathway [85]. The profusion of NOTCH1 might predict poor survival and more aggressive behavior in patients with HCC [86, 87]. Both NOTCH1 and NOTCH4 were immunohistochemical biomarkers predicting HCC patients with short disease specific survival [88]. There was a report suggesting that NOTCH1 functions in endothelial progenitor cells to initiate tumor vasculogenesis in HCC [89]. Activated NOTCH1 expression was strongly associated with HCC metastatic through NOTCH1-Snail-E-cadherin pathway [90]. NOTCH1 and ROS-induced PI3K/AKT pathways cooperatively increased Snail expression and promoted malignancy in HCC [91]. On the other hand, downregulation of Notch signaling activity inhibited HCC invasion by inactivation of matrix metalloproteinase-2 and -9 (MMP-2, -9) and vascular endothelial growth factor (VEGF) [92]. Defective NOTCH signaling led to impaired ability of repairing liver damage [93]. The histone deacetylases inhibitor vaproic acid induced cell growth arrest in HCC via suppressing NOTCH1 and its downstream gene HES1 [94]. NOTCH1 downregulation suppressed the expression of endothelial markers and impaired tube formation. Constitutive NOTCH2 signaling activation played an oncogenic role and induced hepatic tumor formation in mice [95]. Abundant NOTCH2 expression was correlated with anaplasia in human HCC cell lines. The NOTCH2 signaling conferred aggressive behavior and immature morphology to human HCC cells [96]. NOTCH2 was activated in liver cancer stem cells (CSCs) and its activation levels were consistent with clinical severity and prognosis of HCC patients. C8orf4, which attenuated the self-renewal capacity of liver CSCs and tumor propagation, negatively regulated self-renewal of CSCs through suppression of NOTCH2 signaling [97]. Again, self-renewal deficiency and cell growth reduction after NOTCH2 depletion indicated its oncogenic role in HCC [98]. NOTCH3 expression exhibited positive correlation with more aggressive traits and shorter survival in HCC [99]. And it participated in modulating the stemness of tumor cells via inactivation of Wnt/β-catenin pathway [100]. The abundance of NICD3, a symbol of constitutively activated NOTCH signaling, was the only detectable NOTCH3 subunit in HepG2 [101]. Interestingly, despite high NOTCH3 expression, HepG2 showed low NOTCH4 expression [102]. More importantly, NOTCH3 inhibition enhanced the effect of sorafenib by overcoming drug resistance [103]. Limited data manifested that NOTCH4 overexpression might be an independent predictor of short disease specific survival in HCC [88, 102].

#### Colorectal cancer (CRC)

NOTCH1-3 were reported to be oncogenic and highly expressed in human colon adenocarcinomas [104]. Enhanced NOTCH1 was correlated with progression, tumor grade and metastasis resulting from apoptosis inhibition [105, 106]. It also positively regulated the proliferation, colony formation, cell cycling, and tumorsphere formation of human colon cancers [106]. The elevated copy number gain of NOTCH1 together with its mRNA overexpression made it an independent predictor of prognosis in CRC [107, 108]. In colorectal carcinoma cells, NOTCH1-deppendent activation of cell cycle and proliferation were mediated by repression of cyclin-dependent kinase inhibitor p27. Retroviral-transduction activated NOTCH1 resulted in increased expression of stemness related proteins [109]. Moreover, NOTCH1 downregulation significantly sensitized CRC cells to chemotherapy and ionizing radiation [110]. The subcellular localization of β-catenin converged with NICD1 to induce proliferation through the activation of cyclin D1 and HES1 [111]. However, there was a paper uncovered an unexpected suppressive role of NOTCH1 on WNT/β-catenin targeted genes in CRC. Activation of NOTCH1 converted high-grade adenoma into low-grade adenoma in an *APC* ^*min*^ mouse colon cancer model [112]. NOTCH2 expression was decreased in CRC and was associated with tumor differentiation [113]. Negative correlation between NOTCH1 and NOTCH2 was identified in CRC. Increased NOTCH1 expression or decreased NOTCH2 expression represent a risk factor for poor overall survival of CRC patients [114, 115]. NOTCH3 was remarkably up-regulated and promoted tumorigenesis in CRC [116]. Its nuclear expression was related with tumor recurrence and might serve as a novel predictive marker in recurrent CRC patients of stage II and III [117]. Activated NOTCH3 increased MSI-1 level, which was a well established stem cell marker in CRC cells [118]. The miR-1-NOTCH3-Asef pathway was also crucial for CRC cell migration. In this axis, NOTCH3 up-regulated Asef expression and Asef activation was required for colorectal tumorigenesis [119]. miR-206 was another miRNA that potentially regulated NOTCH3 expression in CRC. This miRNA attenuated tumor proliferation and migration through downregulation of NOTCH3 [120].

#### Esophageal cancer

There are two major pathological subtypes of esophageal carcinoma (EC): esophageal adenocarcinomas (EACs) and esophageal squamous cell carcinomas (ESCCs). EACs is considered to arise from a clonal stem like population of cells, in which NOTCH signaling cascade was closely involved [121]. This pathway promoted cell growth and maintained stemness of EAC cells [122]. Moreover, inhibition of NOTCH activity by GSIs decreased tumor growth using patient derived xenograft models [123]. The function of NOTCH1 in ESCC was firstly identified as a tumor suppressor. Activated NOTCH1 inhibited cell proliferation and induced apoptosis in EC9706 [124]. Subsequently, the oncogenic function of NOTCH1 in ESCCs was demonstrated by different groups. NOTCH1 expression was associated with cell aggressiveness and 5-FU drug resistance in ESCC patients [125]. NOTCH1 increased invasion and metastasis of ESCC cell line EC-9706 through EMT transducted by Snail [126]. High NOTCH1 protein expression was related to poor survival in ESCC [127]. Mutually exclusive mutations in NOTCH1 and PIK3CA were identified in ESCCs from Chinese patients with genetic analysis. Mutation in NOTCH1 was related to well-differentiated, early-stage malignancy and less metastasis to regional lymph nodes [19]. NOTCH3 contributed to esophageal cell fate decisions by promoting squamous cell differentiation while preventing dedifferentiation to mesenchymal cell lineages expressing ZEBs, through which inhibition of NOTCH pathway promoted TGF-β-mediated EMT [128].

#### Pancreatic cancer (PC)

In human samples, Notch pathway components were highly expressed in pancreatic adenocarcinoma. Targeting Notch signaling pathway by natural agents eliminates pancreatic CSCs, which suggested a treatment of patients with PC [129]. Ectopic NOTCH activation induced accumulation of nestin-positive precursor cells and expansion of metaplastic ductal epithelium, which was identified as precursor lesion for PC [130]. Moreover, some reports suggested Notch mediated tumor-initiating effects by expanding undifferentiated precursor cells through TGFα. Inhibition of Notch pathway by GSIs reduced PC cell growth. In invasive PCs, NOTCH1 and downstream targets such as HES1 were up-regulated in lesions varying from tubular complexes to carcinoma [131]. Moreover, a profusion of NOTCH1 resulted in induction of EMT phenotype [132]. In PC cells, the tyrosine kinase c-Src directly mediated NOTCH1 and Furin interaction, which regulated carcinogenesis and cancer cell growth [133]. Downregulation of NOTCH1 inhibited proliferation, increased apoptosis, reduced cell migration and invasion of PC cells [134]. However, NOTCH1 exerted tumor suppressor function in a model of K-RAS-induced pancreatic ductal adenocarcinoma [135]. NOTCH2 was highly expressed in ductal cells and pancreatic intraepithelial neoplasia lesions (PanIN) using genetically engineered mice. Conditional ablation of NOTCH2 slowed down PanIN progression and delayed survival time through Myc signaling inhibition [136]. Nuclear accumulation of NOTCH3 was observed in pancreatic adenocarcinomas, which was associated with adverse clinical features and correlated with STAT3 overexpression and phosphorylated AKT [137]. Suppression of NOTCH3 inhibited cell proliferation and decreased PI3K/AKT activity in PC [138].

### The Notch signaling pathway in other solid tumors

The Notch pathway has been implicated in breast cancer development. One mechanism was to develop the adenocarcinomas through pathway activation and the other mechanism was the Numb expression loss [139]. NOTCH signaling also governed the self-rehabilitation of breast cancer stem cells [140]. Its activity has been shown to induce metastasis of breast cancer cells to bone [141]. NOTCH overexpression involved in breast carcinogenesis through inhibition of apoptosis [142]. Moreover, increased Notch signaling was sufficient to transform normal breast epithelial cells through suppression of apoptosis [142]. Different NOTCH receptors played different roles in breast cancer. NICD1 was accumulated in breast cell cells comparing with normal tissue. Elevated NOTCH1 were noted in poorly differentiated tumors, while higher NOTCH2 levels were correlated with more differentiated tumors [143, 144]. Overexpression of NOTCH1/4 active forms altered both normal human and murine mammary epithelial cells [145, 146]. In breast cancer, NICD1 drove mammary tumorigenesis in mice through the target gene Myc [147]. NOTCH2 signaling increased apoptosis, whereas NICD4 promoted cell proliferation and growth in MDA-MB-231 cells [5].

The first evidence of Notch oncogenic role in lung cancer was identified in a tumor-associated translocation between chromosome 15 and 19. NOTCH3 was located in chromosome 19 nearing the breakpoint and suggested to be over-expressed in lung cancer [148]. Deregulation of Notch pathway was a relatively frequent event in none-small cell lung carcinoma (NSCLC). Activation of Notch pathway by either NOTCH1 upregulation or Numb downregulation occurred in 30% primary human NSCLCs [149]. In a transgenic mouse model, activated NOTCH1 was over-expressed in alveolar epithelium and induced alveolar hyperplasia, which was promptly cleared by apoptosis. However, when crossed with Myc-transgenetic mice, the offspring progressed to adenocarcinomas and metastasis [150]. Notch signaling drove proliferation within the lung CSC population [151]. High NOTCH1 expression was significantly correlated with poor outcome in lung adenocarcinomas [152]. In lung cancer cell lines, NOTCH3 was highly expressed and associated with karyotypic abnormalities [148]. NOTCH3 was frequently co-expressed with EGFR in NSCLC to cooperatively promote tumorigenesis [153, 154]. Therefore, NOTCH3 had crosstalk with the EGFR-mitogen-activated protein kinase pathways resulting in apoptosis inhibition through antiapoptotic protein BIM [155]. By meta-analysis, NOTCH1 and NOTCH3 were correlated with tumor progression and poor prognosis in NSCLC [156].

## Conclusions and future directions

In summary, the functional roles of NOTCH receptors in different cancer types are controversial and targeting NOTCH should depend on cell context. Elucidation of the Notch signaling will help us identify novel targets for anti-cancer drug development.

In GC, the roles of NOTCH receptors is still debatable. These contradictory functions of NOTCH receptors suggest that cellular context is critical to elucidate NOTCH signaling cascade in the pathological process of GC. Multiple issues remain to be adressed in the future study. Firstly, which Notch family member is predominantly expressed in GC should be identified. Secondly, typical Notch signal transduction has been proposed in different cancer types, but the detailed and concrete downstream targets of NOTCH signaling pathway in GC should be comprehensively investigated. Thirdly, in spite of small-molecule inhibitors of the GSI targeting all NOTCH receptors, there is still no any small molecule that efficiently targets a specific NOTCH receptor up to now. More importantly, the effects of targeting NOTCH receptors in clinical practice are not clear at present moment and continued in-depth investigation is required. In summary, the deep understanding of NOTCH receptors will provide better clinical translational potential for GC.

## Additional files

Additional file 1: Figure S1. Methylation status of CpG island within 2000 bp beyond the Transcription Start Site (TSS). (TIF 768 kb)
Additional file 2: Figure S2. Correlation analysis between Helicobacter Pylori (*H. Pylori*) infection and NOTCH1-4 mRNA expression. (TIF 698 kb)
Additional file 3: Figure S3. Correlation between Epstein–Barr virus (EBV) infection and NOTCH1-4 mRNA expression. (TIF 860 kb)

## Abbreviations

CIN: Chromosomal instability
CRC: Colorectal cancer
CSCs: Cancer stem cells
CSL: CBF-1/Suppressor of Hairless/Lag-1
DLL: Delta-like
DSL: Delta/Serrate/LAG-2
EACs: Esophageal adenocarcinomas
EBV: Ebstein-Barr virus
EC: Esophageal carcinoma
EGF: Epidermal growth factor-like
EGFR: Epidermal growth factor receptor
EMT: epithelial-mesenchymal transition
ESCCs: Esophageal squamous cell carcinomas
GC: Gastric cancer
GS: Genomically stable
GSIs: γ-secretase inhibitors
*H. pylori*: *Helicobacter pylori*
HCC: Hepatocellular carcinoma
MMP-2: -9, matrix metalloproteinase-2 and -9
MSI: Microsatellite instability
NICD: NOTCH intracellular domain
NSCLC: none-small cell lung carcinoma
PanIN: Pancreatic intraepithelial neoplasia lesions
PC: Pancreatic cancer
SNPs: Susceptible single nucleotide polymorphisms
T-ALL: T-cell acute lymphoblastic leukemia
TCGA: The Cancer Genome Atlas
VEGF: Vascular endothelial growth factor
