# Supplementary figures and images for: NOTCH receptors in gastric and other gastrointestinal cancers: oncogenes or tumor suppressors?
Source: Mol Cancer. 2016 Dec 9;15:80. doi: 10.1186/s12943-016-0566-7 (PMC5148895; doi:10.1186/s12943-016-0566-7)

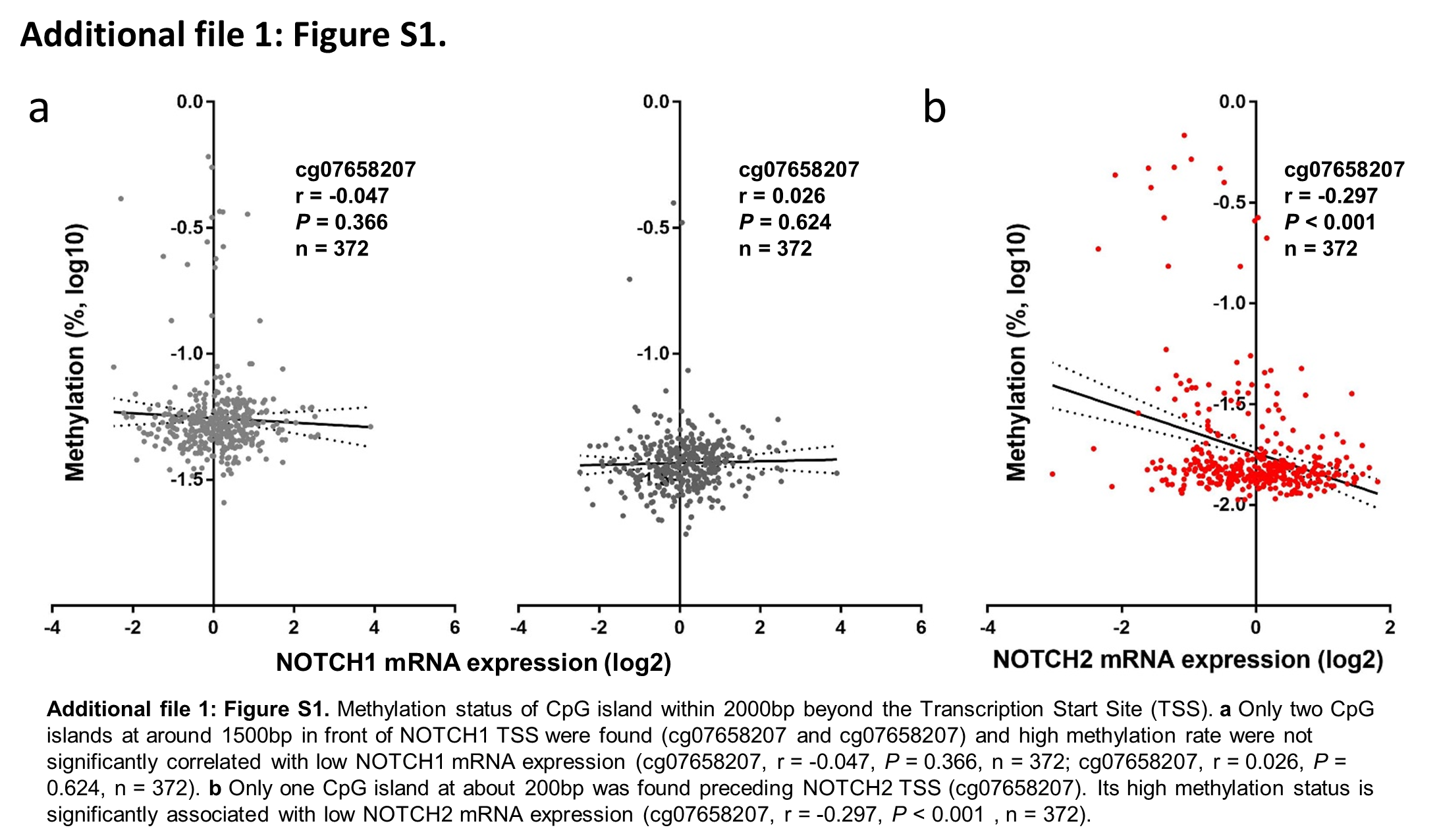

Supplement: Additional file 1: Figure S1. — Methylation status of CpG island within 2000 bp beyond the Transcription Start Site (TSS). (TIF 768 kb) [file 12943_2016_566_MOESM1_ESM.tif]

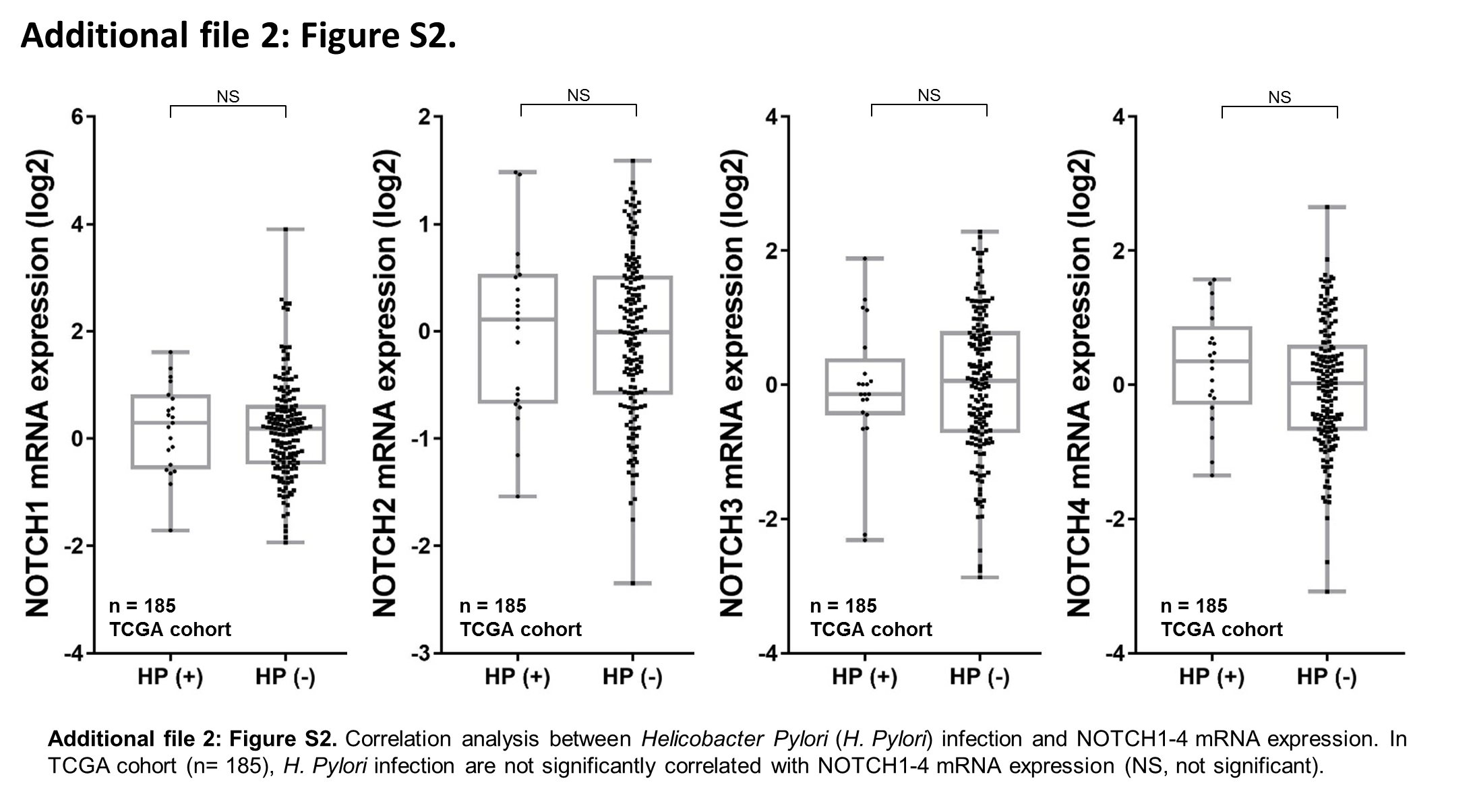

Supplement: Additional file 2: Figure S2. — Correlation analysis between Helicobacter Pylori (H. Pylori) infection and NOTCH1-4 mRNA expression. (TIF 698 kb) [file 12943_2016_566_MOESM2_ESM.tif]

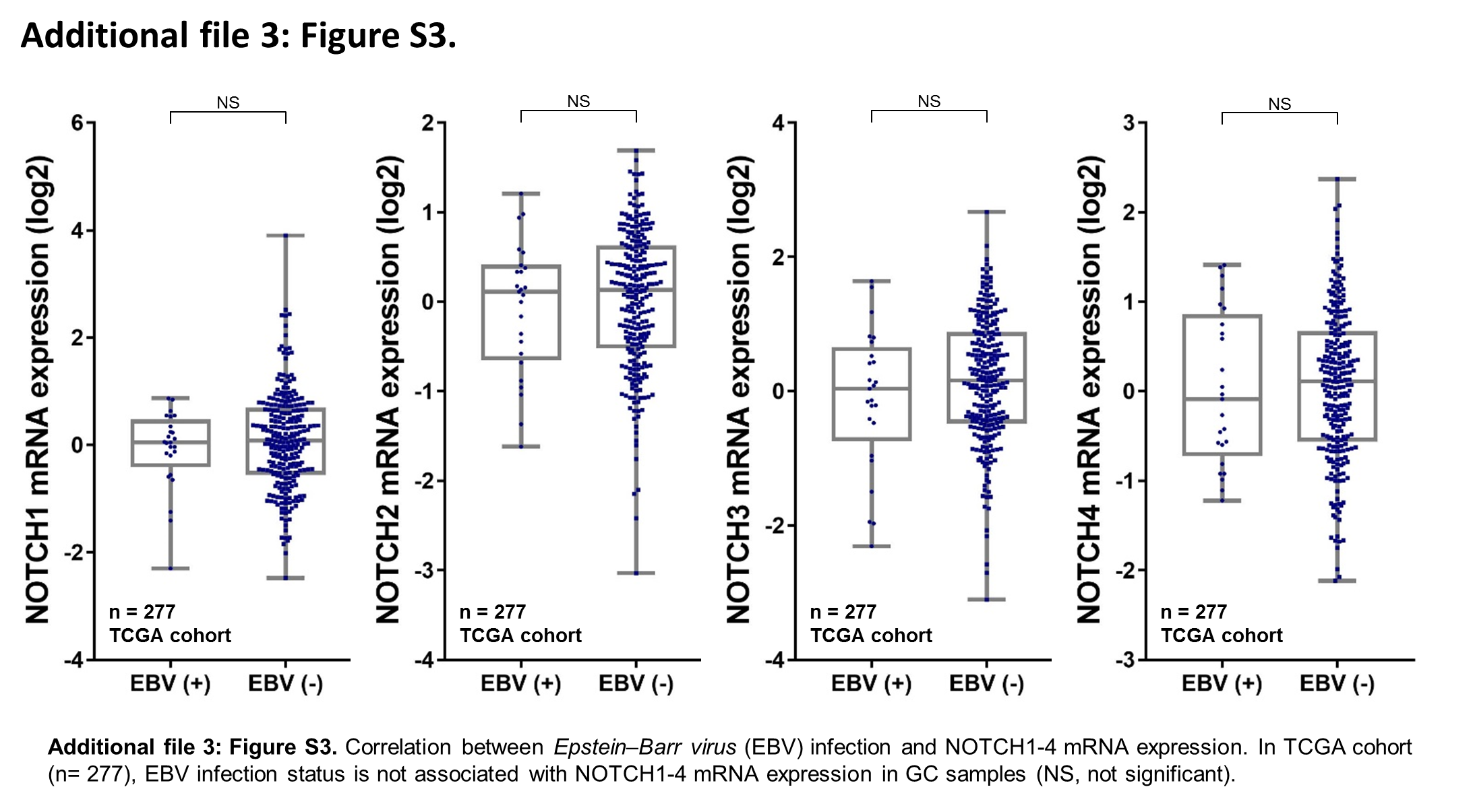

Supplement: Additional file 3: Figure S3. — Correlation between Epstein–Barr virus (EBV) infection and NOTCH1-4 mRNA expression. (TIF 860 kb) [file 12943_2016_566_MOESM3_ESM.tif]
